# Seasonal Dynamics of Algae-Infecting Viruses and Their Inferred Interactions with Protists

**DOI:** 10.3390/v11111043

**Published:** 2019-11-09

**Authors:** Sandra Gran-Stadniczeñko, Anders K. Krabberød, Ruth-Anne Sandaa, Sheree Yau, Elianne Egge, Bente Edvardsen

**Affiliations:** 1 Department of Biosciences, University of Oslo, P.O. Box 1066 Blindern, 0316 Oslo, Norway; a.k.krabberod@ibv.uio.no (A.K.K.); elianne.egge@uni-due.de (E.E.); bente.edvardsen@ibv.uio.no (B.E.); 2 Department of Biological Sciences, University of Bergen, 5020 Bergen, Norway; ruth.sandaa@uib.no; 3 Department of Marine Biology and Oceanography, Institute of Marine Sciences (CSIC), 08003 Barcelona, Spain; syau@icm.csic.es

**Keywords:** *Phycodnaviridae*, *Mimiviridae*, metagenomics, Oslofjorden, viral-host co-occurrences

## Abstract

Viruses are a highly abundant, dynamic, and diverse component of planktonic communities that have key roles in marine ecosystems. We aimed to reveal the diversity and dynamics of marine large dsDNA viruses infecting algae in the Northern Skagerrak, South Norway through the year by metabarcoding, targeting the major capsid protein (MCP) and its correlation to protist diversity and dynamics. Metabarcoding results demonstrated a high diversity of algal viruses compared to previous metabarcoding surveys in Norwegian coastal waters. We obtained 313 putative algal virus operational taxonomic units (vOTUs), all classified by phylogenetic analyses to either the *Phycodnaviridae* or *Mimiviridae* families, most of them in clades without any cultured or environmental reference sequences. The viral community showed a clear temporal variation, with some vOTUs persisting for several months. The results indicate co-occurrences between abundant viruses and potential hosts during long periods. This study gives new insights into the virus-algal host dynamics and provides a baseline for future studies of algal virus diversity and temporal dynamics.

## 1. Introduction

Virus-like particles (VLPs) are widely spread throughout the world’s oceans and are considered to be highly abundant (approximately 1 × 10^7^ viruses mL^−1^), genetically diverse, and dynamic components of the planktonic community [1]. As viruses follow host abundance, total viral abundance is highest in productive coastal waters (~10^8^ viruses mL^−1^) decreasing offshore and deeper in the water column [2,3,4].

Viruses play a major role in marine ecosystems. They are substantial agents of mortality in marine microbial communities, influencing species distribution and abundance and maintain the coexistence of competing species [5,6]. Consequently, viruses are involved in the transformation of organic matter between microbial biomass and the dissolved organic carbon pool [7,8]. The release of organic matter through the viral shunt has been estimated to be 150 billion tons of organic carbon per year [3]. A strong correlation between viral activity and carbon export by sinking particulate matter has recently been observed [9,10,11,12], indicating that viruses significantly contribute not only to the structuring of host communities, nutrient release, and ecosystem productivity, but also to the downward vertical transport of particulate carbon that comprises the biological carbon pump [2].

Most described viruses infecting algae are large double-stranded DNA (dsDNA), often referred to as giant viruses due to their large genome size (>200 kb) and icosahedral virion structure. They belong to two families: the *Phycodnaviridae* [13,14] and the *Mimiviridae* [15]. These two families, together with the *Asfarviridae*, *Iridoviridae*, and *Poxiviridae* make up a monophyletic group [16] referred to as Nucleo-Cytoplasmic Large DNA Viruses (NCLDV [17]). All viruses within the *Phycodnaviridae* infect algae, while members of the *Mimiviridae* infect both photosynthetic and non-photosynthetic protists. Viruses infecting photosynthetic protists all fall within one subcluster within the *Mimiviridae* family, e.g., viruses infecting the haptophytes *Phaeocystis pouchetii*, *Prymnesium kappa*, and *Haptolina ericina* [18,19], while those members infecting heterotrophic protists are polyphyletic [15,20]. Even though the taxon richness of protist-infecting viruses may exceed that of prokaryotes and archaea in the ocean [21], very few genetically characterized taxa with a known host of these two families are available to date.

Studies of algal viruses suggest that they may be species-specific or strain-specific to their host, but many different viruses can infect the same species [19,22,23]. Algal viruses infecting different species have, however, also been described [19]. One approach for measuring these interactions is by correlating viral diversity and abundance with host diversity and abundance to better understand the underlying mechanisms driving the dynamics. Unfortunately, there is no universal marker gene, like the 16S or 18S, common to all viruses [24]. Nevertheless, certain marker genes can be used that capture specific groups of viruses, like the major capsid protein (MCP) of dsDNA algal viruses [25,26,27,28]. By targeting this group of viruses both acute boom-bust infections, where a specific virus can lyse a dense bloom of host cells within hours, and more persistent infections where viruses and hosts can coexist, have been described [26,29]. The latter can be explained by viral resistance, immunity, and/or strain specificity, or by the virus becoming less virulent [30,31,32,33].

In order to expand our knowledge of marine algal virus ecology we have conducted a one-year metabarcoding study of algal viral diversity and dynamics in the Northern Skagerrak, South Norway. We addressed the following questions: 1) Which algal viral OTUs (Operational Taxonomic Units) are dominating the Outer Oslofjorden and can they be linked to characterized algal viruses? 2) How is the algal virus community composition and relative abundance changing over the year? 3) How do the algal viruses co-occur with various co-existing protists? 4) Can co-occurrence analyses give new information about potential virus-algal host relationships?

## 2. Materials and Methods

### 2.1. Sampling

Sea-water samples from the Outer Oslofjorden were collected monthly at the OF2 monitoring station (59.17 N, 10.69 E) between March 2010 and June 2011 except for July 2010, when sampling was not performed. The samplings were performed at the same time of the day each month. Niskin bottles (4 × 5 L) attached to a CTD (conductivity, temperature, depth) rosette were used to collect water samples from a depth of 1 m (subsurface) for virus and protist collection (20 L). The water for virus samples were first filtered through a 200 µm mesh sieve to remove zooplankton and then through a 0.45 µm pore-size, low-protein binding, Durapore membrane filters of 142 mm in diameter (Millipore) mounted in a steel tripod (Millipore, Billerica, USA) by peristaltic pumping (Masterflex, Cole-Parmer, IL, USA). Further, samples were concentrated (~10 psig, high speed) to a final volume of 50 mL using the QuixStand benchtop system with hollow 100,000 pore size fiber cartridges (NMWC) and stored in sterile falcon tubes. Then, 10 × 250 µL of the virus concentrate were transferred into sterile cryo-tubes, flash-frozen in liquid nitrogen, and stored at −80 °C until further processing. Water for protist samples were filtered as described previously [34].

### 2.2. High-Throughput Sequencing

Viral DNA extraction, purification, and PCR amplifications of a part of the MCP gene were done as described in detail in [26], with the exception for PCR re-amplification, where a 2.5 µL template was used. The targeted fragment length of the MCP gene from known viruses ranged ca 347–518 bp [35]. A total of 4 replicates from each sample were then pooled and purified with an AMPure XP bead purification kit (Beckman Coulter, Brea, USA). A pooled sample of 75 µL with a concentration of 22.5 ng µL^−1^ of DNA, quantified by Nanodrop, was sent for sequencing. Library preparation and Lib-A unidirectional amplicon sequencing was performed on 20 µL of the DNA sample (11 ng µL^−1^) in a Roche 454 GS-FLX Titanium (Microsynth AG, Balgach, Switzerland). A total of 203,229 viral reads were obtained.

For the protist samples, nucleotide extraction was performed as described in Gran-Stadniczeñko et al. [34]. Shortly, RNA was extracted and amplified using RNA NucleoSpin II (Macherey-Nagel, Düren, Germany). RNA was then reverse-transcribed to cDNA with the High-Fidelity first Strand cDNA Synthesis Kit (Agilent, Santa Clara, CA) with random primers. A PCR of cDNA was performed using the eukaryote specific primers by Stoeck and co-workers TAReuk454FWD1 and TAReuk-REV3 (see [34]). Amplicon library was generated as specified by Roche (Basel, Switzerland) and sequenced with the 454 GS-FLX Titanium platform at the Norwegian Sequencing Centre (NSC), University of Oslo.

### 2.3. Bioinformatic Pipeline

Viral and protist 454 reads were processed through the QIIME v.1.9.1 pipeline [36]. AmpliconNoise v.1.6.1 and Perseus [37] were used to correct errors of the 454 reads and removed putative chimeras. Clustering of reads into vOTUs (viral OTUs) and pOTUs (protist OTUs) was performed using UCLUST v1.2.22 [38] with a 97% sequence similarity for viruses and 98% for protists.

A total of 582 vOTUs were obtained based on 127,348 reads. Putative spurious data (single singletons, non-algal virus OTUs, and vOTUs that could not be aligned, possibly non-MCP genes), were removed leaving a total of 313 vOTUs. Subsampling was then performed to the minimum read number of 1696 with the “rarefy” option in the Vegan package in R [39]. A complete description of the pOTUs can be found in Gran-Stadniczeñko et al. [34].

### 2.4. Taxonomic Classification and Phylogenetic Analyses

A first taxonomic assignation of the vOTU nucleotide sequences was done with BLASTn against the Viral GenBank database in ViroBLAST with default parameters [40]. In addition, a phylogenetic placement of vOTUs was performed, based on the amino acid (aa) sequence of the vOTUs. Representative nucleotide sequences from the vOTUs were translated into aa with GeneMark [41,42,43]. When the aa sequence predicted by GeneMark was shorter than the expected length based on the nucleotide sequence (i.e., the aa sequence was less than one third the length of the nucleotide sequence) they were manually inspected. The manual inspection consisted of aligning the nucleotide sequence to the closest hit in GenBank and checking for mutations, insertions, and deletions leading to premature stop codons or frameshifts in the predicted aa sequences. These differences in the nucleotide sequence are most likely due to sequencing errors. Sequences that had wrongly inserted stop codons or frameshifts were manually corrected and translated into an aa sequence. The resulting aa sequences were aligned against a previously published reference alignment [26] of 15 *Mimiviridae* and 16 *Phycodnaviridae* viruses, the closest hits in ViroBLAST, and the top hits from the NCBI non-redundant protein database [44]. The top hits from the non-redundant protein database was determined using blastp in Geneious version 10.2.3 [45].

Alignment construction started with sequences longer than 200 aa using MAFFT L-ins-i [46,47]. The shorter fragments were then added to the reference alignment using the *addfragment* algorithm which takes shorter sequences and adds it to an alignment, keeping the gaps, and the relative position of the characters in the original alignment [48]. Ambiguously aligned positions were removed with trimAl using the *gappyout* setting [49]. This alignment consisted of 403 virus sequences. A second alignment consisting of only the 22 most abundant vOTUs and the sequences of 15 *Mimiviridae* and 16 *Phycodnaviridae* from [26] was constructed with the same approach as the larger dataset. Phylogenetic trees were constructed for both alignments with FastTree2 [50], implemented in Geneious 10.2.3 [45], and visualized in FigTree v1.4.3 [51].

### 2.5. Network Construction

Association networks were constructed to analyze the co-occurrence of algae and viruses. The dataset was prepared for network construction after first filtering OTUs not present in at least 2 of the samples. The dataset consisted of 342 pOTUs recently described in [34], and 42 vOTUs. Co-occurrence networks were constructed using *SparCC* [52] as implemented in the R package *SpiecEasi* [53]. *SparCC* was run with default settings and with 500 bootstraps. Two-sided pseudo p-values were calculated for both datasets independently and edges with a p-value of >0.05 and a correlation score of <|0.5| were deleted. Protist-protist associations were also deleted, since we were primarily interested in the associations between viruses and protists. The visualization of the networks was done with *Cytoscape* v3.6.1 [54].

## 3. Results

We assessed the *Phycodnaviridae* and *Mimiviridae* taxonomic composition, vOTU richness, and relative abundance through the year by high-throughput amplicon sequencing (metabarcoding) of 15 samples taken monthly from the Outer Oslofjorden, Skagerrak, Southern Norway. The conserved MCP gene was amplified from all samples and resulted in 126,775 reads of 200–400 bp in length, representing 313 putative algae-infecting vOTUs after filtration, with a 97% similarity clustering, the removal of singletons, and translation into amino acids (aa). The vOTUs were named according to decreasing relative abundance before subsampling. After subsampling to the lowest number of reads per sample (1696 reads), to be able to compare samples, 243 vOTUs remained. None of the rarefaction curves for the 15 samples reached a plateau, indicating that the sequencing depth was not sufficient to capture the full diversity at Outer Oslofjorden (Figure S1). The most abundant vOTU (vOTU 1) contained 53% of all reads. A total of 22 vOTUs had more than 50 reads (0.2% of total reads per vOTU) representing 95% of the total reads and were considered the most abundant vOTUs, whereas 208 vOTUs had each less than 0.1% of the reads (Table S1).

### 3.1. Virus Diversity

Of the 313 vOTUs, 95 had a similarity ≥90% to reference sequences obtained by BLASTn against Viral Genbank on the ViroBLAST platform (Table S1). Of these, 36 had the best matches to cultured viral sequences. The putative hosts were two haptophytes (*Haptolina ericina* and *Haptolina. hirta*) and four prasinophytes (*Micromonas pusilla*, *Micromonas* sp. strain RCC1109, *Ostreococcus lucimarinus*, and *O. mediterraneus*). A total of 59 vOTUs best matched uncultured environmental MCP viral sequences. The rest had a lower similarity to cultured or uncultured reference sequences, meaning they had not been found elsewhere by metabarcoding.

**Figure 1:**
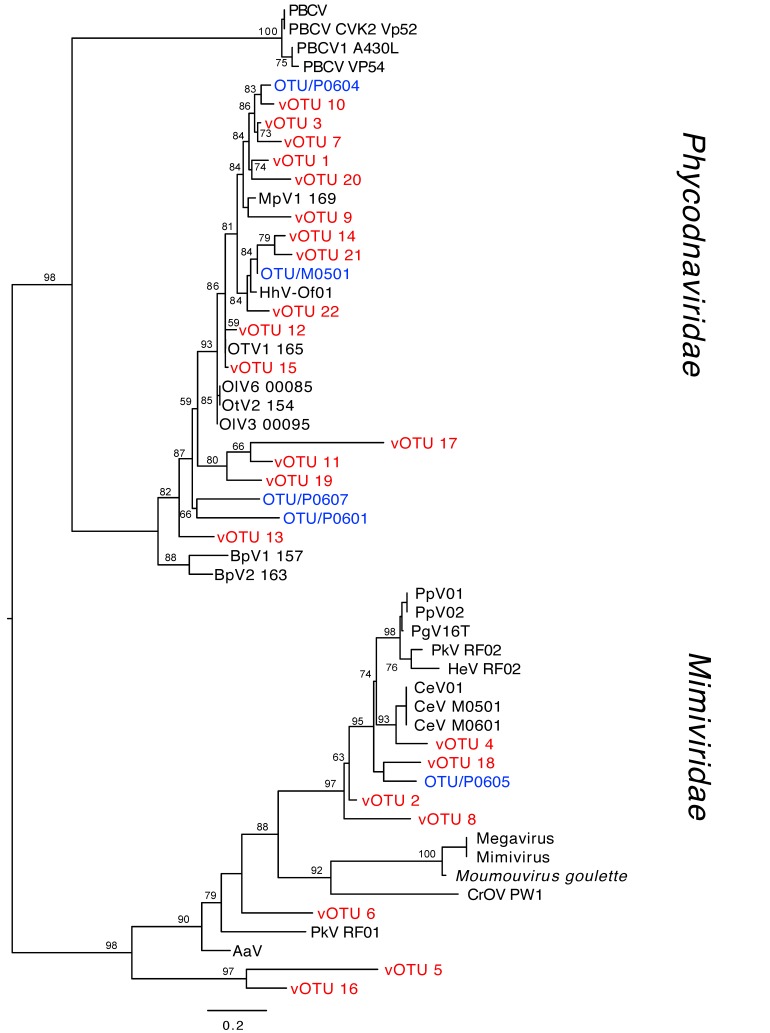
Phylogenetic tree based on relevant *Phycodnaviridae* and *Mimiviridae* major capsid protein (MCP) reference sequences (black), virus Operational Taxonomic Units (OTUs) found in a previous study in Raunefjorden (blue [35]), and the 22 most abundant viral OTU (vOTU) algal viruses (>50 reads) from Outer Oslofjorden (red). Bootstrap values >50 are shown at the nodes.

*Phycodnaviridae* and *Mimiviridae* phylogenetic trees based on MCP amino acid vOTU sequences were constructed including all 313 vOTUs (Figure S2, File S1), and 22 of the most abundant vOTUs (>50 reads per vOTU, Figure 1, File S2). The table with accession numbers of reference sequences is presented in Table S2. A total of 54 vOTUs were taxonomically placed within the *Mimiviridae* and 259 within the *Phycodnaviridae* families. Several clades in both families did not cluster with any reference sequence and some vOTUs only clustered with uncultured environmental MCP sequences (Figure S2, File S1). Several uncultured environmental reference sequences that originally were classified as *Phycodnaviridae* were placed in the *Mimiviridae* clade. Fifteen of the 22 most abundant vOTUs were placed in the *Phycodnaviridae* family and seven in *Mimiviridae* (Figure 1). The two most abundant *Phycodnaviridae*, vOTU 1, and vOTU 3 clustered together with vOTU 7, vOTU 10, and vOTU 20, close to an environmental vOTU from Puddefjorden (Western Norway, OTU/P0604 [35]). They were also placed in the same major clade as vOTU 9 and the sequence of a virus infecting the picoplankton chlorophyte *Micromonas pusilla* (MpV1) with a bootstrap value of 84%. Three vOTUs (vOTU 14, 21, and 22) clustered with the environmental OTU/M0501 from Raunefjorden (Western Norway [35]) and a virus infecting *Haptolina hirta* (HhV-Of01) with high bootstrap values (>80%). Furthermore, vOTU 12 and 15 clustered with sequences from a virus infecting *Ostreococcus tauri* (OtV1 165). The most abundant *Mimiviridae* vOTUs were vOTU 2 and 4. The vOTU 4 clustered with *Chrysochromulina ericina* virus (CeV, infecting *Haptolina ericina*) with a high bootstrap support (95%), whereas vOTU 2 did not cluster with any known viral reference sequence. Furthermore, a new branch consisting of two vOTUs (vOTU 5 and 16) was obtained as a sister to the *Mimiviridae* family (Figure 1).

### 3.2. Temporal Variation

**Figure 2:**
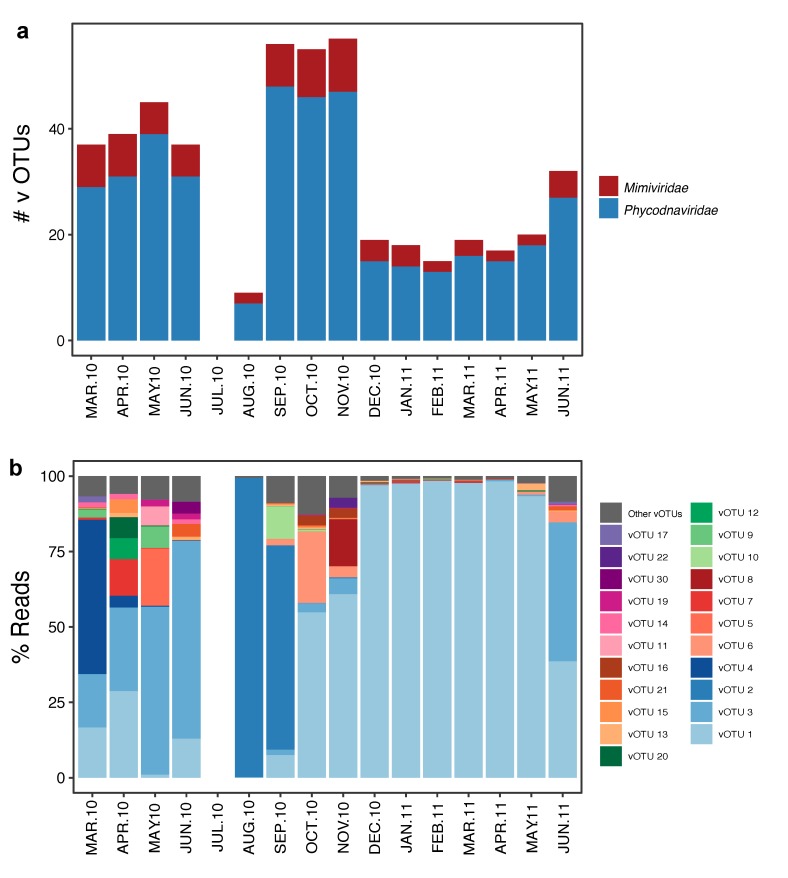
Barplots representing: (**a**) Number of vOTUs per family and (**b**) proportional abundance (percentage reads) of the most abundant vOTUs (>50 reads per vOTU) over time at Outer Oslofjorden station.

Virus richness was highest from September to November 2010 and lowest in August 2010 (~55 and 9 vOTUs respectively, Figure 2a and Figure S3) whereas the Shannon diversity index was highest in April 2010 and lowest in August 2010 (2.06 and 0.07, respectively, Figure S3). The *Phycodnaviridae* family was more diverse than the *Mimiviridae* in all samples (Figure 2a), but *Mimiviridae* dominated in abundance in March, August, and September 2010, and was negligible from April 2010 to June 2010 and from December 2010 to April 2011 (Figure S4). Among the 22 most abundant vOTUs, six were highly frequent, present in ten or more samples (vOTU 1, 3, 7, 12, 13, and 15), whereas four were only present in one sample (vOTU 8, 11, 22, and 30, Table S1, Figure 2b). The *Phycodnaviridae* vOTU 1, which clustered close to MpV1, dominated for eight months from October 2010 to May 2011 (Figure 2b). The *Phycodnaviridae* vOTU 3, also clustering close to MpV1, dominated in May and June 2010 and in the June 2011 samples. The *Mimiviridae* vOTUs 2 and 4 dominated in the March, August, and September 2010 samples and were rare or absent in other samples.

**Figure 3:**
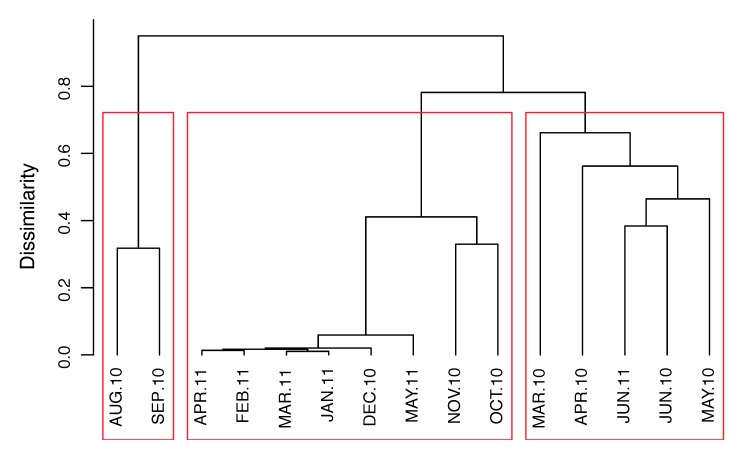
Bray clustering analyses representing Bray–Curtis similarities in virus community composition based on vOTU abundances.

Hierarchical clustering of samples based on Bray–Curtis dissimilarities showed three well-distinguished clusters. The August and September 2010 samples significantly differed from the other 13 (Figure 3). All samples between December 2010 and May 2011 formed a cluster with highly similar samples that may be explained by the strong dominance of vOTU 1 in these samples (Figure 2b).

### 3.3. Temporal Variation of Viruses and their Potential Hosts

**Figure 4:**
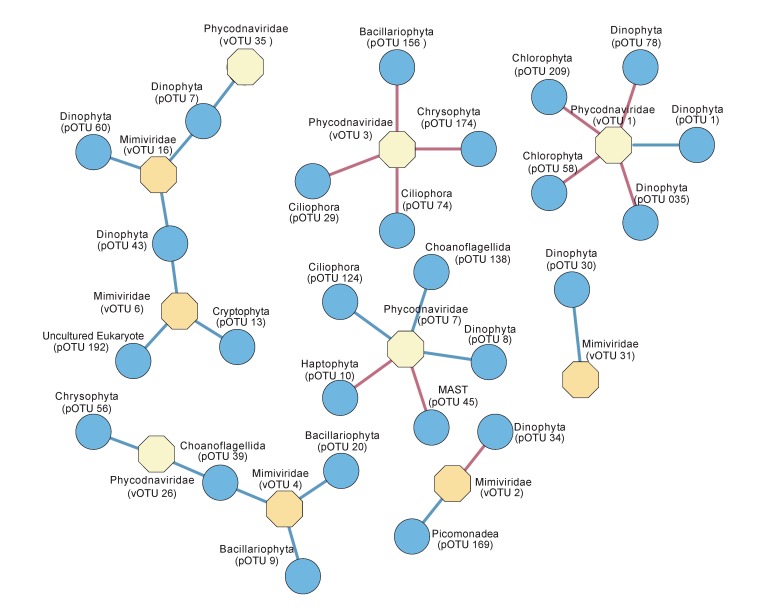
Network analysis showing the co-occurrence between virus and protist taxa (represented as blue and beige nodes respectively). Lines between nodes indicate positive (blue) and negative (red) SparCC correlation >|0.5| (two-sided pseudo p-value < 0.05) between the abundances of linked taxa. Network was visualized by Cystoscape V3.3.0.

Co-occurrence network analyses were done to detect positive and negative protists-virus correlations between our protist [34] and virus datasets (Figure 4) from Outer Oslofjorden. A positive correlation between two OTUs means they show the same pattern of sequence abundance between samples, thus they co-occur. A negative correlation means that they have an opposite pattern of sequence abundance across samples. Networks with a correlation score >|0.5| and p < 0.05 showed no clear positive or negative patterns for *Phycodnaviridae* and *Mimiviridae* with pOTUs. The *Phycodnaviridae* vOTU 1, which clustered with a virus infecting the chlorophyte *Micromonas pusilla* (MpV1), had negative correlations with two chlorophyte pOTUs that had a best match to *Pycnoccocus provasolii*. (pOTU 58) and *Pyramimonas* sp. (pOTU 209), together with two dinoflagellate pOTUs (Figure 4, Table 1), as well as a positive correlation with the most abundant protist, *Karenia papillonaceae* (pOTU 1). The *Mimiviridae* vOTU 2, which did not cluster with a known reference sequence, had a positive correlation with a picobilizoan (heterotrophic picoplankton) and a negative correlation with an uncultured dinoflagellate. The *Phycodnaviridae* vOTU 3 only presented negative correlations with two ciliates, one diatom, and one chrysophyte. vOTU 4, which clustered close to the *Chrysocromulina ericina* virus CeV, did not show any significant correlation with a haptophyte pOTU, but showed positive co-occurrences with two diatoms and a choanoflagellate (heterotrophic opisthokont protist). Furthermore, the *Phycodnaviridae* vOTU 7, showed negative correlations with a MAST (uncultured heterotrophic stramenopile), and the haptophyte *Emiliania huxleyi*, as well as positive correlations to two uncultured alveolates and a choanoflagellate. The remaining *Phycodnaviridae* and *Mimiviridae* vOTUs did not show significant correlations with potential protist hosts.

**Figure 5:**
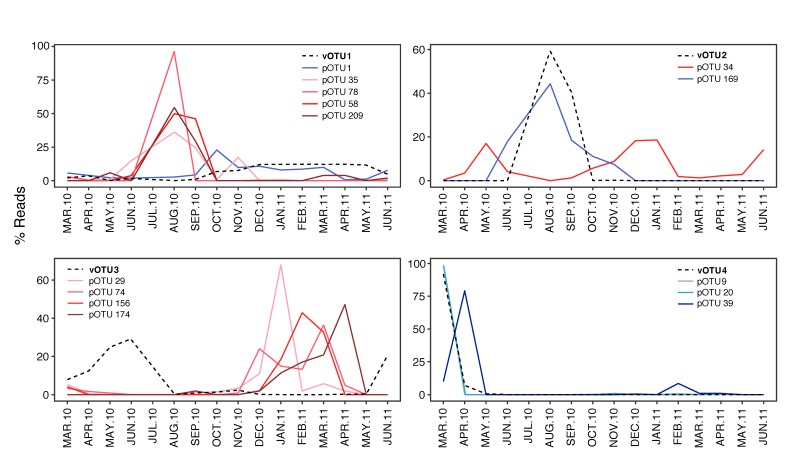
Temporal distribution of the four most abundant viruses, vOTU1-4, and their co-occurring protists. Virus OTU relative abundances are represented with dotted lines, those of pOTUs with continuous lines. Red lines indicate negative correlations between virus and protists, blue lines indicate positive correlations.

The seasonal dynamics of protists at the Outer Oslofjorden OF2 sampling campaigns have been described in [34]. Figure 5 shows the temporal pattern between the most abundant viruses and their co-occurring protist from Figure 4. Protist and viruses with positive correlations showed similar temporal distributions, whereas protist and viruses with negative correlations showed opposite temporal patterns. The protist with negative correlations with vOTU 1 showed a similar temporal pattern, with its highest abundances in July and August.

**Figure 6:**
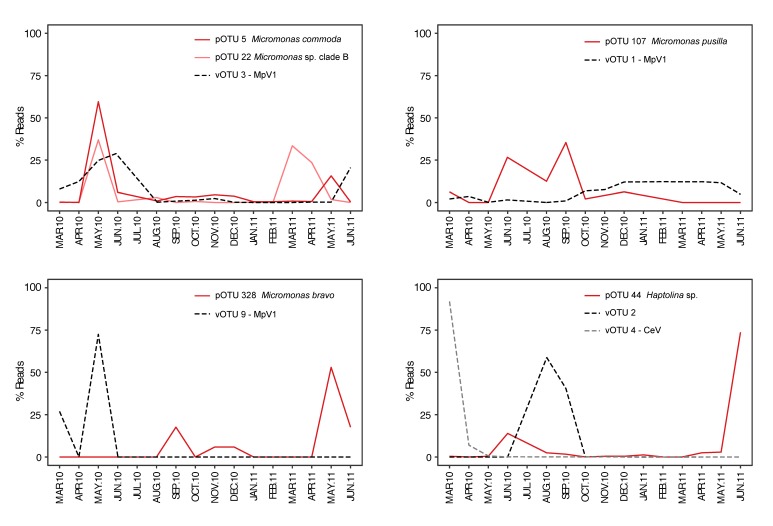
Relative abundances over time of vOTUs matching known viruses (MpV1 and CeV) and their possible host *Micromonas commoda* and *M*. sp. clade-B-subarctic, *M. pusilla*, *M. bravo*, and *Haptolina* sp. obtained by Gran-Stadniczeñko et al. [34].

Combining the distribution pattern of the most abundant vOTUs assigned to a cultured virus with a known host, with that of their putative protist host ([34], Figure 6) shows a temporal variation for all vOTUs and pOTUs. For *M. commoda*, *M.* sp. clade-B-subarctic, *M. pusilla*, and *Haptolina* sp., the vOTUs occurrence occasionally overlapped with that of the hosts’ and peaked after a drop of the hosts’ abundances, whereas no overlap between abundances of *Micromonas bravo* and MpV1 was observed.

## 4. Discussion

### 4.1. Viral diversity

This is the first metabarcoding study on algal viruses from the Skagerrak and Oslofjorden. Here we have studied viruses in seawater passing a 0.45 µm pore-size filter, which include both free, non-cellular viruses and those within host cells that might have been disrupted during filtration. Using MCP gene metabarcoding we revealed a relatively high diversity of algal viruses compared to previous studies in Norwegian coastal waters [26]. In the previous study, however, a clustering level of 95% was used, which might mask some of the diversity by including more variation in the same vOTU. The MCP gene is a commonly used marker for algal viruses, but the primers are known to not amplify the totality of dsDNA MCP diversity, such as EhV and phaeovirus [35]. Based on the MCP gene, we obtained 313 vOTUs, all classified by phylogenetic analyses to either the *Phycodnaviridae* or *Mimiviridae* families with high bootstrap support (>60%). A total 12% (36 vOTUs) had a best match with BLASTp to a cultured algal virus, whereas 70% (218 vOTUs) had 89% or a lower similarity to any available virus sequences. Furthermore, by phylogenetic placement, the majority of the vOTUs formed clades without any reference sequence. This is in accordance with previous studies, e.g., by Clerissi et al. [55], pinpointing the lack of characterized algae-infecting viruses and the need for more cultured and characterized reference strains. Several of the uncultured vOTUs have been classified as *Phycodnaviridae* but were in our study placed in the *Mimiviridae* clade: ABU23699, ABU23704 [35], AHN92263, AHN92262, AHN92288, AHN92249 [56], and AGI16567 [57]. Originally, all dsDNA icosahedral viruses infecting photosynthetic protists were first classified as *Phycodnaviridae*. Recent phylogeny based on Nuclear Cytoplasmic Large DNA Viruses (NCLDV) orthologous genes, however, showed that many of the large dsDNA algal viruses belong to the *Mimiviridae* (references in [15]). The taxonomy of algal viruses is at present under revision by the ICTV. A revision of the taxonomy of algal virus reference sequences in gene databases will therefore be needed once a new approved classification is available.

Of the 22 most abundant vOTUs, seven were assigned (by phylogeny and BLAST, ≥90% similarity) to cultured marine viruses infecting different protist taxa such as *Micromonas pusilla* virus (MpV1), *Haptolina hirta* virus (HhV-Of01), *Ostreococcus* spp. viruses (OtV, OlV, and OmV viruses), and *Chrysochromulina ericina* viruses (CeV, *Haptolina ericina* was previously named *Chrysochromulina ericina)*. Members of the genera *Micromonas* and *Haptolina* are common phytoplankton species in the Oslofjorden [26,34,58]. *Micromonas pusilla* was until recently the only described species of this genus, but was divided into several species by Simon et al. [59]. *Micromonas commoda* was the most abundant *Micromonas* species in Outer Oslofjorden OF2 station, but *M. pusilla*, *M. bravo*, and *M*. sp. clade-B-subarctic were also detected [34]. We found several vOTUs assigned to MpV1, which may turn out to have different *Micromonas* species as their hosts. The picoplanktonic prasinophyte genus *Ostreococcus* has a wide distribution in temperate to tropical marine waters, but was not recorded by metabarcoding north of 60°N by Tragin and Vaulot [60]. The vOTUs 12 and 15 were in our phylogenetic analysis clustering with the cultured and genome characterized strain OtV-165, isolated from the English Channel and infecting *Ostreococcus tauri*, strain OTH95 [61]. With BLAST, however, vOTUs 12 and 15 had a best match to the OmV1 virus, infecting *Ostreococcus mediterraneus*. Due to this incongruence, we classified vOTU 12 and 15 as *Ostreococcus* sp. virus (Table S1). Both *O. tauri* and *O. mediterraneus* were recorded for the first time from Outer Oslofjorden station OF2 by Gran-Stadniczeñko et al. [34], and are thus potential hosts of these viruses, despite previously having been found mostly in warmer waters [60].

### 4.2. Succession of Viral OTUs and Potential Hosts

The viral community at Oslofjorden showed a clear temporal variation, but not a recurring seasonal pattern as has been demonstrated in other studies (e.g., [62]). The lack of seasonality could be due to methodological limitations such as primer pairs targeting only a small fraction of the algal virus population or PCR biases towards the amplification of certain genotypes. A too low sampling frequency should also be considered as viral communities change fast (e.g., due to high virus decay rates), and thus boom-bust episodes may be overlooked. A boom-bust episode here refers to a cycle predicted in host and virus population dynamics when host and virus abundances oscillate such that there is a peak in host abundance, followed by a decline that corresponds to a peak in virus abundance. Furthermore, the fact that some pOTUs were not observed in two consecutive samples could be due to different abiotic or biotic factors (e.g., grazing or nutrient availability), and not to viral lysis. Therefore, we may need closer sampling intervals (e.g., weekly or even daily) to catch a direct link between host and virus [63]. Another explanation may be the interannual variation of the host community structure. Algal viruses may require a certain density of the host for infection, which may vary from year to year (see review by Short [64]).

The newly described species *Micromonas commoda*, recently separated from *Micromonas pusilla* [59], showed the highest relative abundance during May 2010 and then decreased abruptly in the following months [34]. Our temporal comparisons with potential hosts showed that the vOTU 1 (clustering with MpV1) had an opposite pattern compared to *M. pusilla*, which decreased in abundance from September to October 2010, at the same time as the vOTU 1 was gradually increasing in abundance. The MpV1-like vOTU 1 was the most abundant vOTU and dominated for eight months. Furthermore, vOTU 3 had an affinity to MpV1 and was among the most abundant vOTUs from March to June 2010 and increased in abundance when the potential host *M. commoda* decreased. In a previous study of the *Micromonas pusilla* virus (MpV) in the Skagerrak-Kattegat area, Sahlsten [65] similarly found the highest abundance during spring and lasting for several months. Zingone et al. [66] found likewise the *Micromonas* infecting virus MpV to persist in the water for a long period. Additionally, Zingone et el. [67] found that some strains of *Micromonas* were resistant to viral infection. A coexistence between virus and host during long periods is the most observed pattern in our study, as most of our vOTUs are present at several timepoints (see Figure 2b, Figure 5, and Figure 6) and has also been seen in several other studies [26,27,28]. A persistent relationship is in agreement with the evolution theory on parasite prey interactions (see Opinion by Sandaa and Bratbak [29]) as any ecologically successful parasite will ensure host survival rather than mortality. Viruses may hence be expected to evolve from causing acute infections with high mortality towards less virulent and more persistent infections. Assuming that this theory is correct, most virus-host interactions in nature should be persistent since they have evolved over a long period [68,69]. Another explanation would be that these viruses might infect several similar or even different hosts, allowing them to proliferate on different host species [19].

In contrast, the most abundant *Mimiviridae* vOTU 2 and 4 appeared to dominate during shorter periods or specific time-points. This pattern is generally described for lytic viruses and suggests that these *Mimiviridae* viruses have a boom-bust relationship with their host, causing acute infections and killing nearly all host cells. This viral-host interaction can be observed once or twice during the study period. Johannessen et al. [26] found a similar temporal distribution pattern with some algal virus OTUs highly abundant only at specific time-points, although most viral OTUs were persistent. Most studies of algal viruses and their hosts have focused on the boom-bust system, probably as these are easier to detect.

Finally, the temporal variation in abundance of co-occurring viruses and protists (Figure 5) explains our network results. vOTU 1 (MpV1), for instance, had a positive relationship with pOTU 1 assigned to *Karenia papillonaceae* in addition to four negative correlations to photosynthetic taxa (two chlorophytes and two dinoflagellates) that varied similarly through the year. These positive/negative correlations do not necessarily imply that the viruses are able to infect such diverse hosts, but that they may influence one host group which in turn affect the growth conditions for others. Alternatively, the protist groups show similar or opposite responses to other environmental factors than viruses.

## 5. Conclusions

All vOTUs could be classified to either *Phycodnaviridae* or *Mimiviridae* based on their major capsid protein amino acid sequence. *Phycodnaviridae* showed both the highest richness and relative abundance. We obtained 313 vOTUs and a majority did not match with any cultured or environmental sequences available in gene databases (70% at a ≥90% similarity). A total of 12% of the vOTUs had an affiliation to a known cultured virus with either a putative prasinophyte or haptophyte algal host. These hosts have all been found in the Outer Oslofjorden. The viral dynamics show a clear temporal variation with some vOTUs persisting for several months, suggesting they co-exist with their hosts, whereas others were present at specific time points, suggesting a possible boom and bust relationship with their hosts. The co-occurrence analysis could not with any certainty reveal new host relationships, but suggests some relationships to be examined in future studies. The comparison between the relative abundance of viruses and of their potential hosts can give new insight into the virus-algal host dynamics and the ecological role of algal viruses. This study also provides a baseline for future studies of algal virus diversity and temporal dynamics. With more cultured and genetically characterized viruses, more of the viruses reported here may be connected to a host and contribute to a better understanding of the ecological importance of algal viruses and their distribution in space and time.

## Figures and Tables

**Table 1:** Details on positive and negative co-occurrences between virus MCP gene OTUs (vOTUs) obtained in this study and protist 18S rRNA gene OTUs (pOTUs) obtained by Gran-Stadniczeñko et al. [34].

VIRUS				PROTIST			
vOTU ID	Family	Lowest Taxonomic Level	Co-Occurrence^1^	pOTU ID	Supergroup	Group	Lowest Taxonomic Level
**vOTU 1**	Phycodnaviridae	*Micromonas pusilla* virus (MpV1)	+	**pOTU 1**	Alveolata	Dinophyta	*Karenia papillonaceae*
			-	**pOTU 35**	Alveolata	Dinophyta	MALV-III
			-	**pOTU 78**	Alveolata	Dinophyta	Uncultured dinoflagellate
			-	**pOTU 58**	Archaeplastida	Chlorophyta	*Pycnococcus provasolii*
			-	**pOTU 209**	Archaeplastida	Chlorophyta	Pyramimonadales
**vOTU 2**	Mimiviridae	Uncultured	-	**pOTU 34**	Alveolata	Dinophyta	Uncultured dinoflagellate
			+	**pOTU 169**	Picozoa	Picomonadea	Picobiliphyta
**vOTU 3**	Phycodnaviridae	*Micromonas pusilla* virus (MpV1)	-	**pOTU 74**	Alveolata	Ciliophora	Cyclotrichia
			-	**pOTU 29**	Alveolata	Ciliophora	Strombidiidae
			-	**pOTU 156**	Stramenopila	Bacillariophyta	Coscinodiscophyceae
			-	**pOTU 174**	Stramenopila	Chrysophyta	Chrysophyceae-Clade-C
**vOTU 4**	Mimiviridae	*Chrysochromulina ericina* virus (CeV)	+	**pOTU 39**	Opistokonta	Choanoflagellida	Stephanoecidae-Group-D
			+	**pOTU 20**	Stramenopila	Bacillariophyta	*Pseudo-nitzschia multiseries*
			+	**pOTU 9**	Stramenopila	Bacillariophyta	*Thalassiosira nordenskioeldii*
**vOTU 6**	Mimiviridae	Uncultured	+	**pOTU 13**	Hacrobia	Cryptophyta	*Teleaulax gracilis*
			+	**pOTU 192**	Uncultured eukaryote	Uncultured Eukaryote	Uncultured eukaryote
**vOTU 7**	Phycodnaviridae	*Micromonas pusilla* virus (MpV1)	+	**pOTU 124**	Alveolata	Ciliophora	Haptoria
			+	**pOTU 8**	Alveolata	Dinophyta	*Gyrodinium helveticum*
			-	**pOTU 10**	Hacrobia	Haptophyta	*Emiliania huxleyi*
			+	**pOTU 138**	Opistokonta	Choanoflagellida	Stephanoecidae-Group-D
			-	**pOTU 45**	Stramenopila	MAST	MAST-1C
**vOTU 16**	Mimiviridae	Uncultured	+	**pOTU 7**	Alveolata	Dinophyta	*Lepidodinium chlorophorum/L. viride*
			+	**pOTU 60**	Alveolata	Dinophyta	MALV-III
			+	**pOTU 43**	Alveolata	Dinophyta	Uncultured dinoflagellate
**vOTU 26**	Phycodnaviridae	Uncultured	+	**pOTU 39**	Opistokonta	Choanoflagellida	Stephanoecidae-Group-D
			+	**pOTU 56**	Stramenopila	Chrysophyta	Chrysophyceae-Clade-C
**vOTU 31**	Mimiviridae	*Chrysochromulina ericina* virus (CeV)	+	**pOTU 30**	Alveolata	Dinophyta	Uncultured dinoflagellate
**vOTU 35**	Phycodnaviridae	*Micromonas pusilla virus* (MpV1)	+	**pOTU 7**	Alveolata	Dinophyta	*Lepidodinium chlorophorum/L. viride*

^1^ “+“ positive correlations, “-“ negative correlations.

## Supplementary Materials

The following are available online at https://www.mdpi.com/1999-4915/11/11/1043/s1, Figure S1: Rarefaction curves for the MCP reads at the different samples obtained at Outer Oslofjorden. Figure S2: Phylogenetic tree based on available Phycodnaviridae and Mimiviridae MCP reference sequences (black), vOTUs found in a previous study in Raunefjorden (blue, [35]) and all algal vOTUs obtained from Outer Oslofjorden (red), available at: https://figshare.com/s/7ff5a7e14308dea9394f. Figure S3: Virus OTU Richness and Diversity over time at the OF2 station. Figure S4: Proportional abundances of Phycodnaviridae and Mimiviridae over time at Outer Oslofjorden. Table S1: MCP vOTUs recorded at station OF2 during the sampling period March 2010 to June 2011 with number of reads in each sample and taxonomic placement. OTUs marked in red were removed after subsampling, available at: https://figshare.com/s/7ff5a7e14308dea9394f. Table S2: Isolated members of the Phycodnaviridae and Mimiviridae families used in Figure 1 study with GenBank accession numbers for the MCP, File S1: Fasta file with MCP AA alignment used for the complete phylogenetic tree presented in Figure S2, available at: https://figshare.com/s/7ff5a7e14308dea9394f. File S2: Fasta file with MCP AA alignment used for the phylogenetic tree with the most abundant vOTUs presented in Figure 1. Available at: https://figshare.com/s/7ff5a7e14308dea9394f.
