# Supplementary material for: Seasonal Dynamics of Algae-Infecting Viruses and Their Inferred Interactions with Protists
Source: Viruses. 2019 Nov 9;11(11):1043. doi: 10.3390/v11111043 (PMC6893440; doi:10.3390/v11111043)
Supplement: Supplementary file 1 [file viruses-11-01043-s001.zip › sup/Table S2 Isolated members.docx]

**Table S2.** Isolated members of the *Phycodnaviridae* and *Mimiviridae* *families* used in Figure 1 study with GenBank accession numbers for the MCP.

| **Virus isolate** | **GenBank accession no.** | **Host species** |
| --- | --- | --- |
| BpV1_157 | [YP_004061587](https://www.ncbi.nlm.nih.gov/protein/YP_004061587.1?report=genbank&log$=protalign&blast_rank=1&RID=ZX6HNXX6015) | *Bathycoccus sp.* |
| BpV2_163 | [ADQ91330](https://www.ncbi.nlm.nih.gov/protein/ADQ91330.1?report=genbank&log$=protalign&blast_rank=2&RID=ZX6HNXX6015) | *Bathycoccus sp.* |
| CeV-M0501 | EU006612 | *Haptolina ericina* |
| CeV-M0601 | EU006613 | *Haptolina ericina* |
| CeV01 | EU006628 | *Haptolina ericina* |
| HeV_RF02 | AHZ86982 | *Haptolina ericina* |
| HhV-Of01 | AHZ86983 | *Haptolina hirta* |
| MpV1_169 | YP_004062052 | *Micromonas pusilla* |
| OlV3_00095 | AFK66095 | *Ostreococcus lucimarinus* |
| OlVG_00085 | AFK65839 | *Ostreococcus lucimarinus* |
| OtV1_165 | YP_003212988 | *Ostreococcus tauri* |
| OtV2_154 | YP_004063587 | *Ostreococcus tauri* |
| PBCV | AB018579 | *Chlorella* sp. |
| PBCV_CVK2 | BAA35143 | *Chlorella* sp. |
| PBCV_VP54 | AB006978 | *Chlorella* sp. |
| PBCV-1 | AAA88828 | *Chlorella* sp. |
| PgV16T | EU006624 | *Phaeocystis globosa* |
| PkV_RF01 | KJ558372 | *Prymnesium kappa* |
| PkV_RF02 | AHZ86984 | *Prymnesium kappa* |
| PpV-01 | EU006631 | *Phaeocystis pouchetii* |
| PpV-02 | EU006623 | *Phaeocystis pouchetii* |
| CrOV-PW1 | YP_003969975BV | [*Cafeteria roenbergensis*](https://www.ncbi.nlm.nih.gov/protein/YP_003970184.1) |
| *Aureococcus anophagefferens* virus | YP_009052173 | *Aureococcus anophagefferens* |
| Megavirus | AGD92382 | Unknown |
| Mimivirus | AGW18172 | Unknown |
| Moumouvirus goulette | AGF85360 | Unknown |
| OTU/M0501 | EU086758 | Unknown |
| OTU/P0601 | EU006616 | Unknown |
| OTU/P0604 | EU006619 | Unknown |
| OTU/P0605 | EU006620 | Unknown |
| OTU/P0607 | EU006622 | Unknown |
