# Supplementary material for: Seasonal Dynamics of Algae-Infecting Viruses and Their Inferred Interactions with Protists
Source: Viruses. 2019 Nov 9;11(11):1043. doi: 10.3390/v11111043 (PMC6893440; doi:10.3390/v11111043)
Supplement: Supplementary file 1 [file viruses-11-01043-s001.zip › sup/Figure S1_Rarefaction curve.pdf]

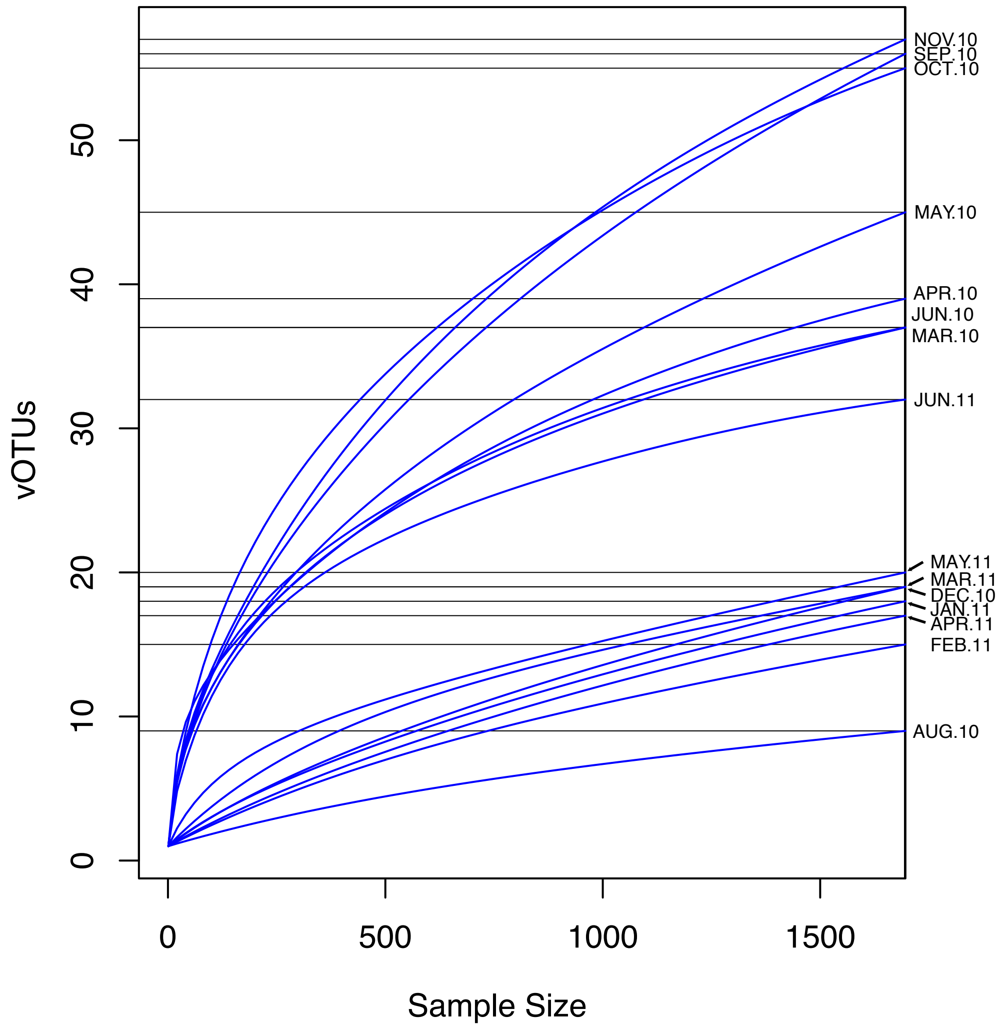

Figure S1: Rarefaction curves for the MCP reads at the different samples obtained at Outer Oslofjorden.
