# Supplementary figures and images for: Seasonal Dynamics of Algae-Infecting Viruses and Their Inferred Interactions with Protists
Source: Viruses. 2019 Nov 9;11(11):1043. doi: 10.3390/v11111043 (PMC6893440; doi:10.3390/v11111043)

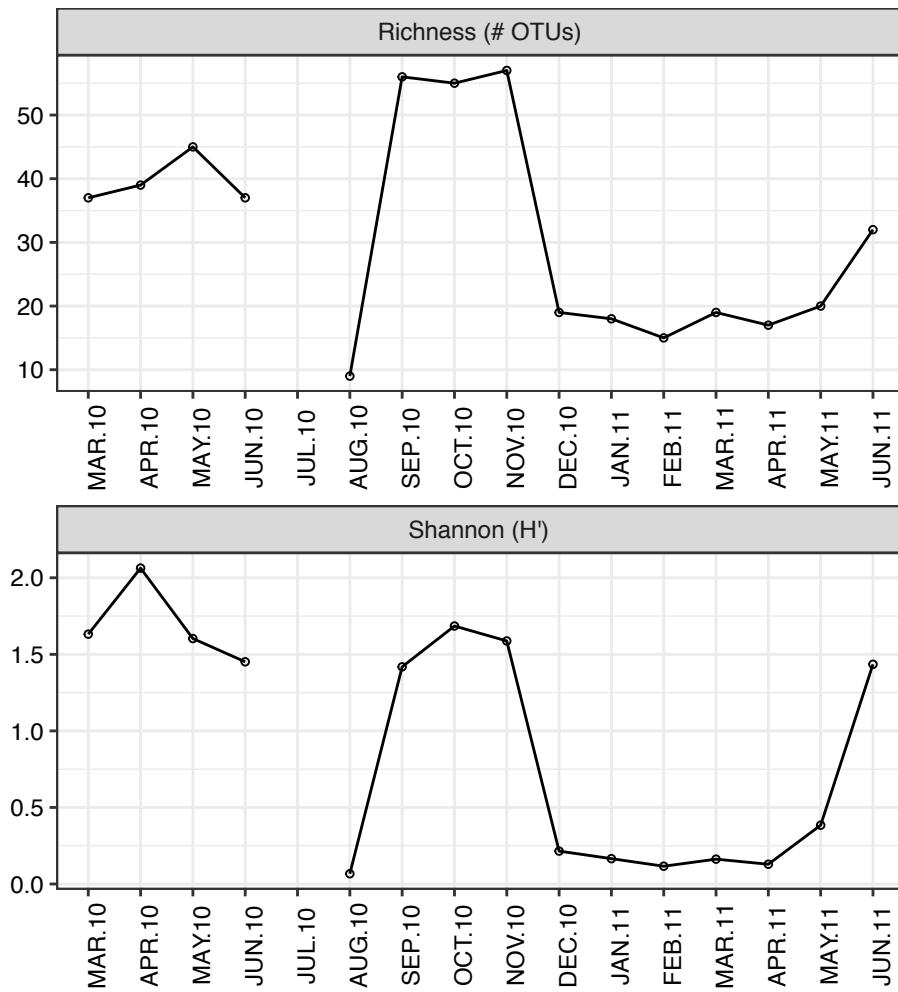

Figure S3: Virus OTU Richness and Diversity over time at the OF2 station

Supplement: Supplementary file 1 [file viruses-11-01043-s001.zip › sup/Figure S3_Diversity.pdf]

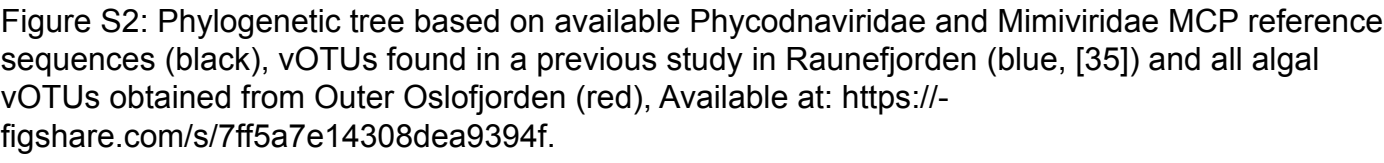

Supplement: Supplementary file 1 [file viruses-11-01043-s001.zip › sup/Figure S2_Full_tree.pdf]
